# Supplementary material for: Gene–gene interaction detection with deep learning
Source: Commun Biol. 2022 Nov 12;5:1238. doi: 10.1038/s42003-022-04186-y (PMC9653457; doi:10.1038/s42003-022-04186-y)
Supplement: Supplementary file 2 — Supplementary Information [file 42003_2022_4186_MOESM2_ESM.pdf]

Supplementary Information "Gene-Gene Interaction Detection with Deep Learning"

Table of contents

Supplementary Note 1 Correlation between genes 2

Supplementary Note 2 Additional simulation studies with complex interactions 3
Supplementary Note 2.1 Compare the power and specificity with different p-value thresholds 3
Supplementary Note 2.2 Simulation studies with only parts of genes interacting 3
Supplementary Note 2.3 Simulation studies with more genes 4
Supplementary Note 2.4 ROC curves 4
Supplementary Note 2.5 PRC curves 5
Supplementary Note 2.6 Ablation studies 9

Supplementary Note 3 Additional simulation studies with simple interactions 10
Supplementary Note 3.1 Simulation studies with only parts of genes interacting 10
Supplementary Note 3.2 Simulation studies with more genes 11

Supplementary Note 4 Additional simulation studies without interactions 12
Supplementary Note 4.1 Calibration plot of existing permutation methods 12
Supplementary Note 4.2 Permutation approach for NNs with maxT correction 12

Supplementary Note 5 Visualization of replicable interactions 13

Supplementary Note 6 Additional experiments on UK Biobank 15
Supplementary Note 6.1 Compare the R^2 of the top-SNP approach and NNs 15
Supplementary Note 6.2 Compare the interaction detection power of the top-SNP approach and NNs . . 15

Supplementary Note 1 Correlation between genes

Shapley values, including Shapley interaction scores, perform poorly on correlated features. Fortunately, the gene representations learned from different SNPs sets are not correlated, because the genes are usually far from each other. In Supplementary Figure 1, we show the correlation between 65 gene representations learned from NNs on UK Biobank and the corresponding 4,322 SNPs.

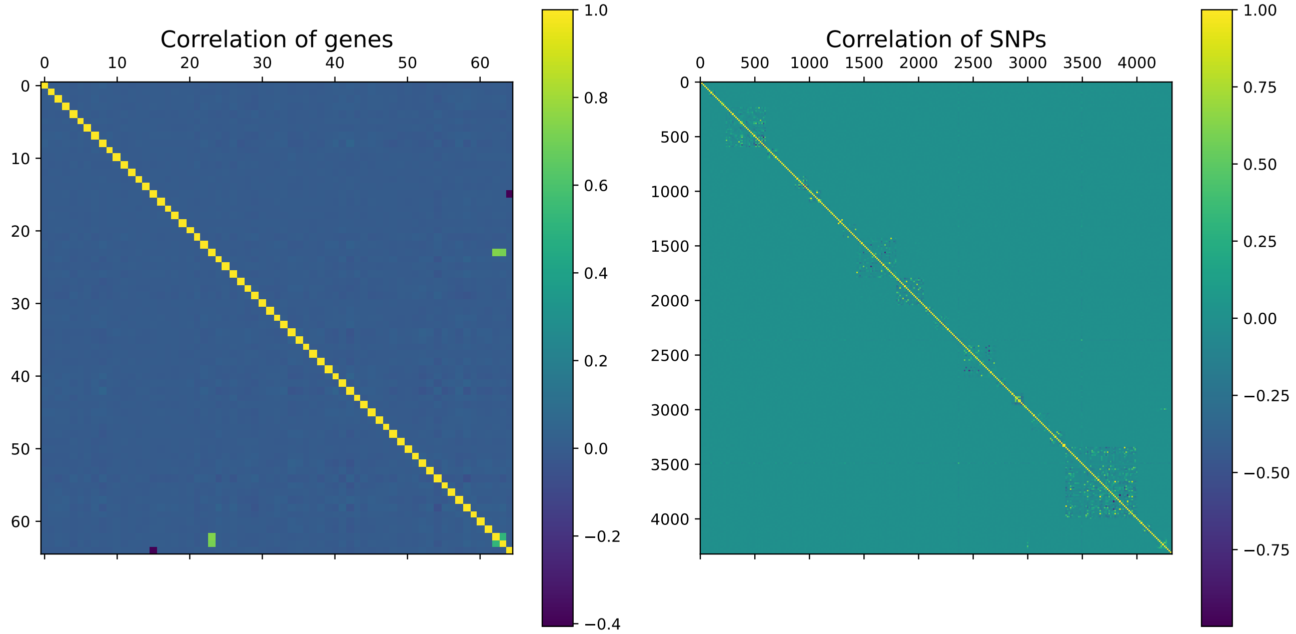

Supplementary Figure 1: Correlation between 65 gene representations (left) and correlation between 4,322 SNPs (right) on UK Biobank experiments. We observe that most genes are not correlated with an averaged absolute correlation to be 0.007. Moreover, we did not observe much high correlation between selected SNPs (averaged absolute correlation is 0.017), because of the LD pruning procedure.

Supplementary Note 2 Additional simulation studies with complex interactions

Supplementary Note 2.1 Compare the power and specificity with different p-value thresholds

In Supplementary Figure 2, we compare the power and specificity of different methods on simulated datasets. We regard interactions are positive if their nominal p-value from permutation is less than 0.02 and 0.1. We notice that by increasing the p-value threshold from 0.02 to 0.1, the power of each method increases, while the corresponding specificity decreases. For NNs, Perm T and Perm R have very low specificity but power 1 in most settings, as they severely underestimate the interaction scores under the null hypothesis and consider all pairwise interactions positive. We observe that although NNs have a specificity similar to or slightly smaller than the other methods, the power of detecting complex interactions is much higher.

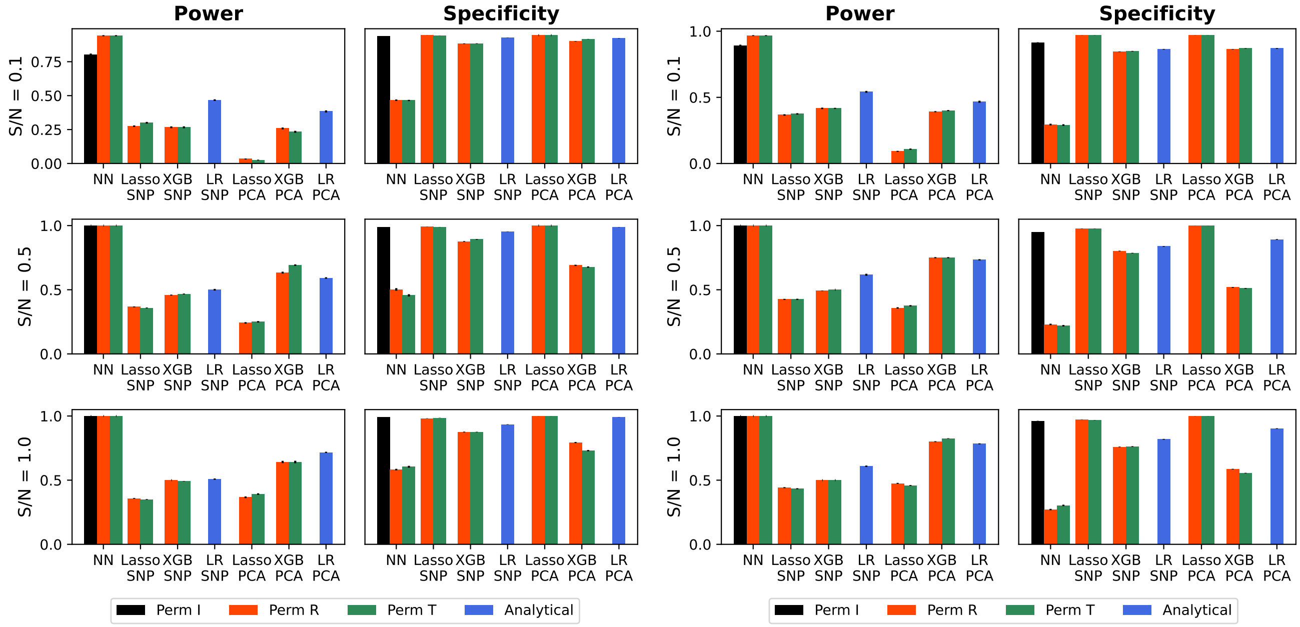

Supplementary Figure 2: Comparison of the power (true positive rate) and specificity (true negative rate) of different methods. We consider interactions to be positive if corresponding p-values are less than 0.02 (left) and 0.1 (right).

Supplementary Note 2.2 Simulation studies with only parts of genes interacting

Here we extend the experiments in Section 2.2.1 by constructing simulation datasets where only a small set of genes have interaction effects but all genes have main effects. We consider two following simulators where only 2 genes and 5 genes out of 10 genes have interaction effects, such that

$$\begin{aligned} \mathbf{g}_i &= \sum_{j=1}^{d_i} \alpha_{ij}\mathbf{x}_{ij},\ \forall i \in \{1, \dots, 10\}, \\ \mathbf{y} &= w_{79} \max\{\mathbf{g}_7, \mathbf{g}_9\} + \sum_{k}^{10} w_k \mathbf{g}_k + \epsilon, \end{aligned} \tag{1}$$

where only the maximum interaction between $\mathbf{g}_7$ and $\mathbf{g}_9$ is retained, and,

$$\begin{aligned} \mathbf{g}_i &= \sum_{j=1}^{d_i} \alpha_{ij}\mathbf{x}_{ij},\ \forall i \in \{1,\dots,10\}, \\ \mathbf{y} &= w_{79}\max\{\mathbf{g}_7,\mathbf{g}_9\} + w_{48}(\mathbf{g}_4-\mathbf{g}_8)^2 + w_{810}\mathbf{g}_8\mathbf{g}_{10} + \sum_{k}^{10} w_k\mathbf{g}_k + \epsilon, \end{aligned} \tag{2}$$

where we keep one interaction for each type, and 5 genes are interacting.

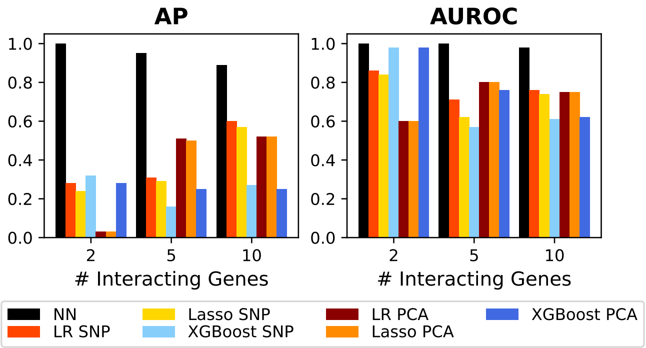

Supplementary Figure 3: Comparison of gene-gene interaction detection methods in terms of AP and AUROC on datasets with complex interactions. We simulate data using S/N = 0.1, data size = 80k, prop causal SNPs = 50%, M/I = 1.0, and three different number of interacting genes: 2, 5, and 10 (simulator shown in the main text). The NN approaches (black bar) are consistently better than existing approaches.

From Supplementary Figure 3, we observe that NNs can detect interactions even better than existing methods when only a small number of genes are interacting in the simulator.

Supplementary Note 2.3 Simulation studies with more genes

Here we scale up the experiments in Section 2.2.1 by considering 50 and 100 genes instead of only 10. We consider the following simulator:

$$\begin{aligned} \mathbf{g}_i &= \sum_{j=1}^{d_i} \alpha_{ij}\mathbf{x}_{ij},\ \forall i \in \{1,\dots,10A\}, \\ \mathbf{y} &= \sum_{a=0}^{A-1} (w_{12}\max\{\mathbf{g}_{10a+1},\mathbf{g}_{10a+2}\} + w_{79}\max\{\mathbf{g}_{10a+7},\mathbf{g}_{10a+9}\} + w_{35}(\mathbf{g}_{10a+3}-\mathbf{g}_{10a+5})^2 \\ &\quad + w_{48}(\mathbf{g}_{10a+4}-\mathbf{g}_{10a+8})^2 + w_{67}\mathbf{g}_{10a+6}\mathbf{g}_{10a+7} + w_{810}\mathbf{g}_{10a+8}\mathbf{g}_{10a+10}) + \sum_{k}^{10A} w_k\mathbf{g}_k + \epsilon, \end{aligned} \tag{3}$$

with $A = 5$ and $A = 10$. In the above simulator, all genes have interaction effects, and the number of interactions are scaled up according to the number of genes.

From Supplementary Figure 4, we observe that as the number of genes of the simulator increases, the performance of all methods drops. This is because the total signal-to-noise ratio is fixed and the effect of each interaction is scaled down when more genes and interactions are included in the simulator. NNs can detect interactions even better than existing methods when the number of genes is high.

Supplementary Note 2.4 ROC curves

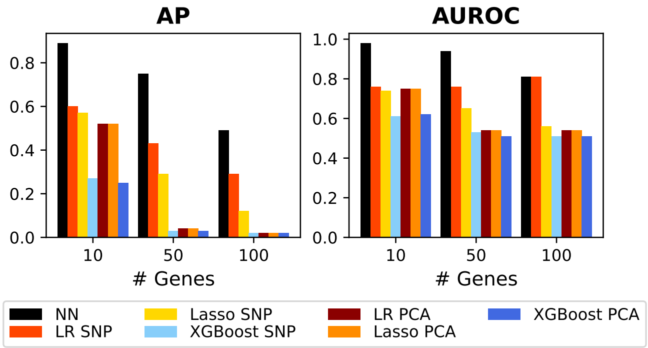

Supplementary Figure 4: Comparison of gene-gene interaction detection methods in terms of AP and AUROC on datasets with complex interactions. We simulate data using S/N = 0.1, data size = 80k, prop causal SNPs = 50%, M/I = 1.0, and three different number of genes: 10 (simulator shown in the main text), 50, and 100. The NN approaches (black bar) are consistently better than existing approaches.

Supplementary Note 2.5 PRC curves

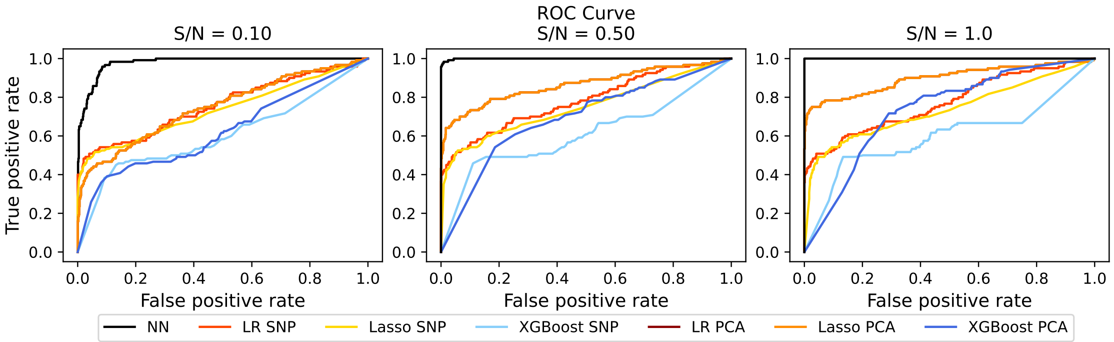

Supplementary Figure 5: The ROC curve for experiments with different S/Ns.

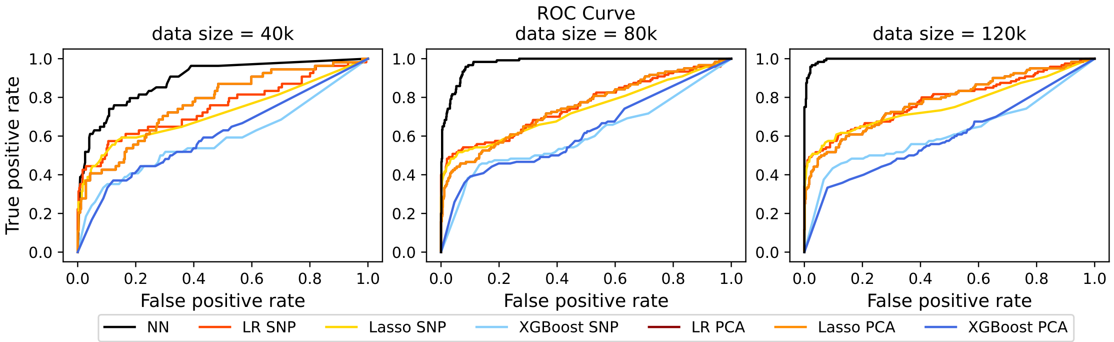

Supplementary Figure 6: The ROC curve for experiments with different training data sizes.

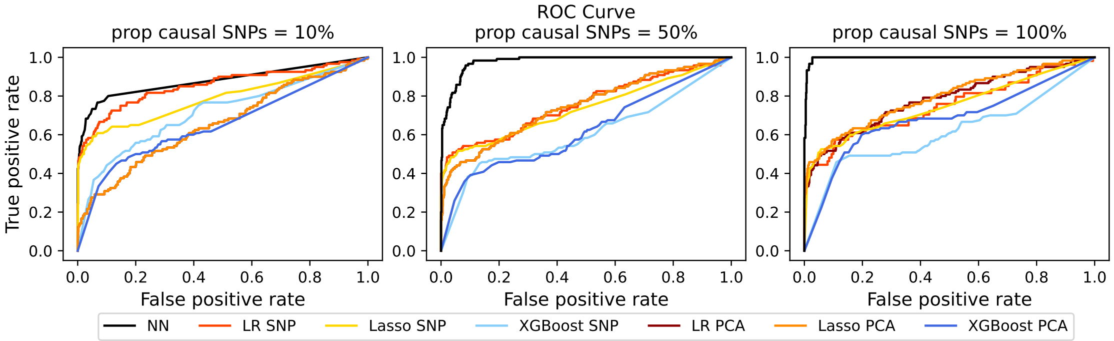

Supplementary Figure 7: The ROC curve for experiments with different proportions of causal SNPs.

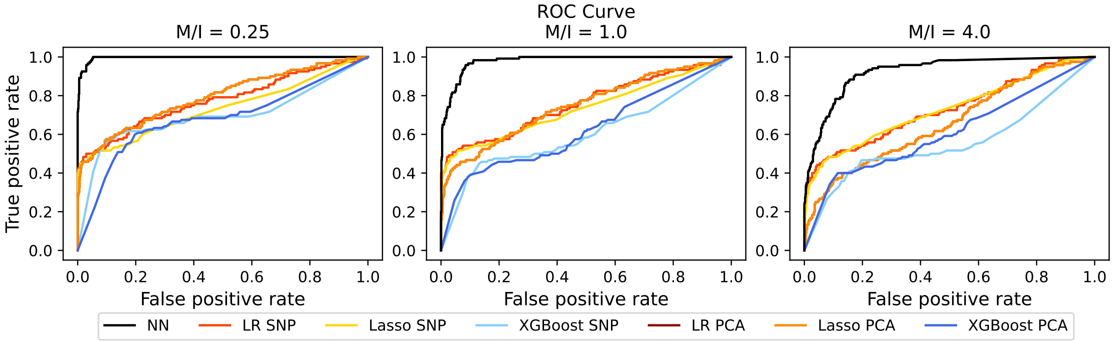

Supplementary Figure 8: The ROC curve for experiments with different main-interaction-ratios.

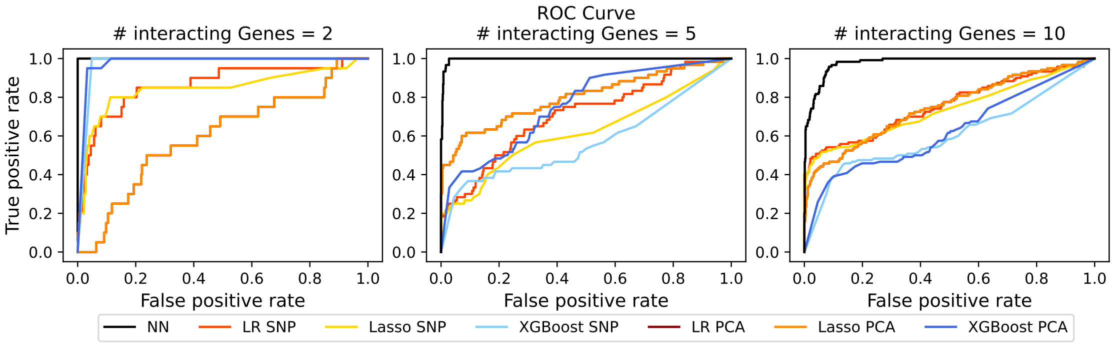

Supplementary Figure 9: The ROC curve for experiments with different number of interactions.

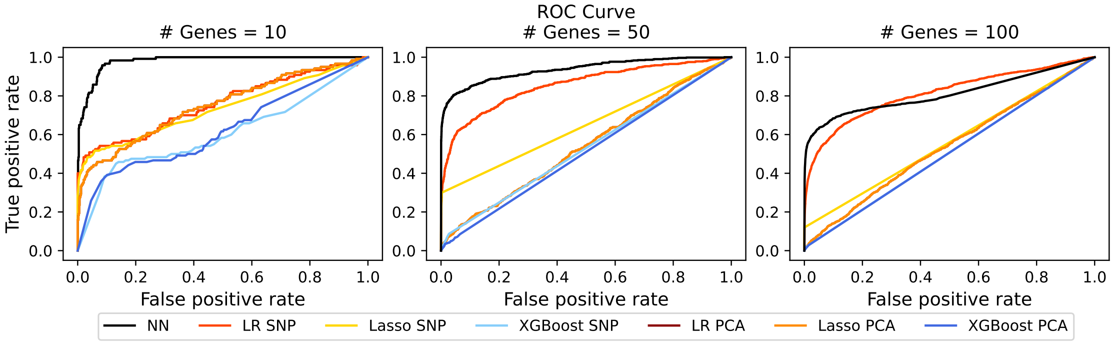

Supplementary Figure 10: The ROC curve for experiments with different number of genes.

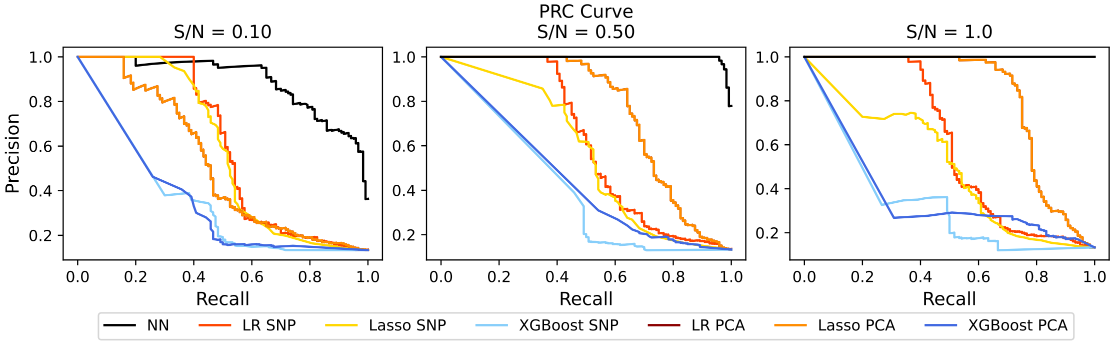

Supplementary Figure 11: The PRC curve for experiments with different S/Ns.

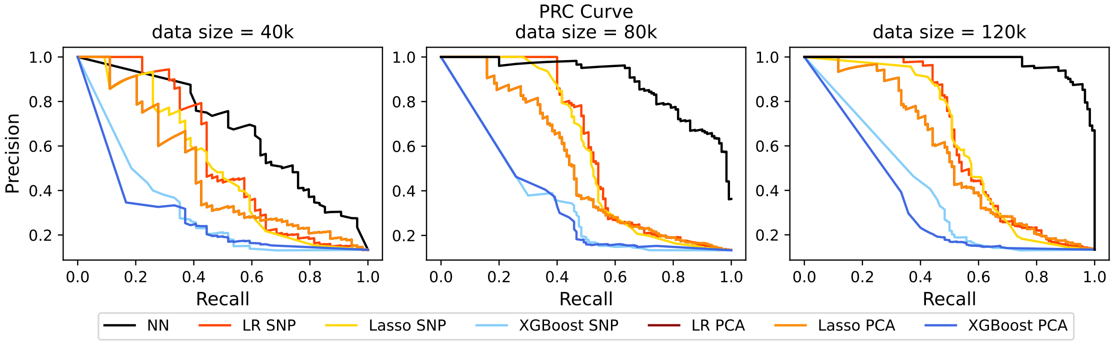

Supplementary Figure 12: The PRC curve for experiments with different training data sizes.

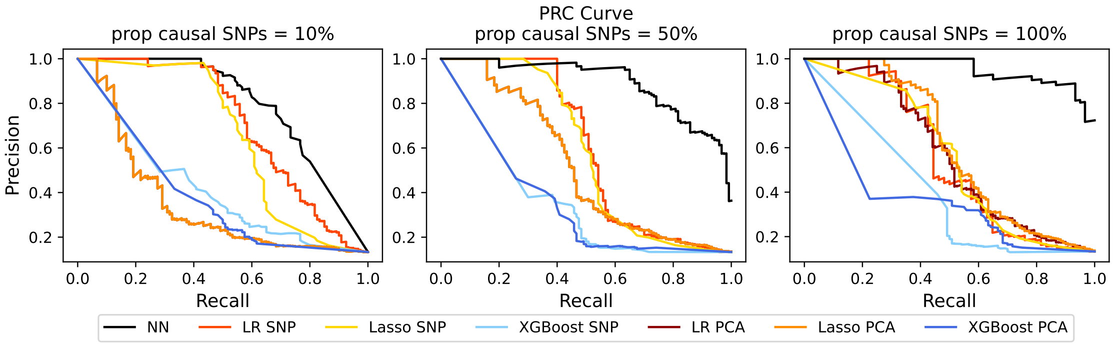

Supplementary Figure 13: The PRC curve for experiments with different proportions of causal SNPs.

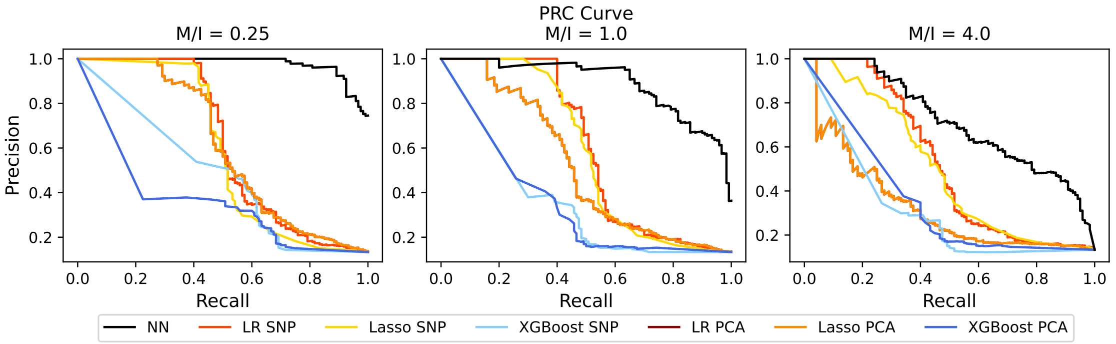

Supplementary Figure 14: The PRC curve for experiments with different main-interaction-ratios.

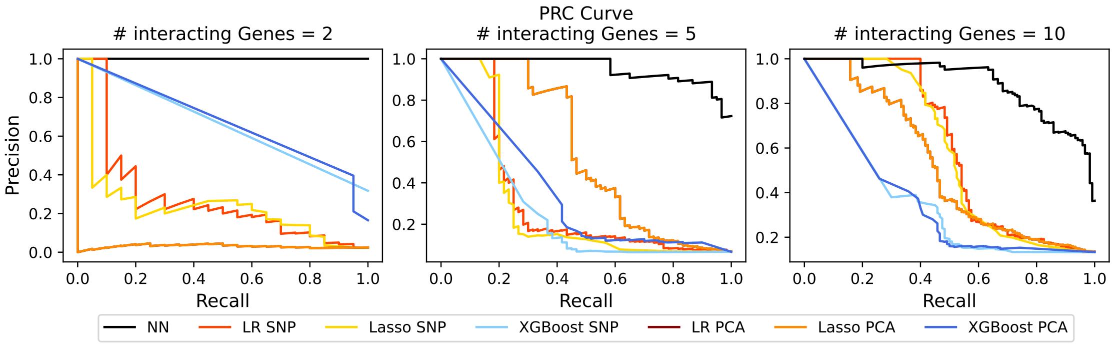

Supplementary Figure 15: The PRC curve for experiments with different number of interactions.

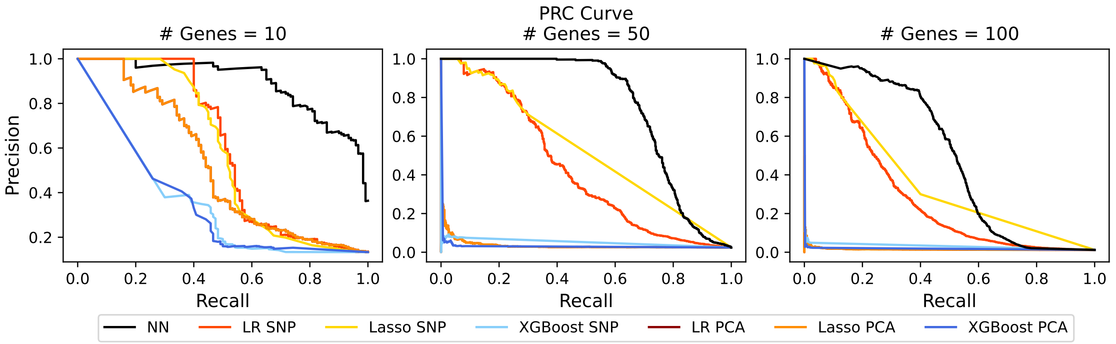

Supplementary Figure 16: The PRC curve for experiments with different number of genes.

Supplementary Note 2.6 Ablation studies

Going from the classical top-SNP regression to the proposed deep learning approach, there are two additional components: 1. $h_{g_i}(\mathbf{X}_i; \mathbf{w}_{g_i})$ (in Equation 6a of the main text) that learns the representation of gene i from all of its SNPs $\mathbf{X}_i$ instead of using the single most correlated SNP as in the top-SNP regression; 2. $h_p(\mathbf{g}_1, \ldots, \mathbf{g}_M; \mathbf{w}_p)$ (in Equation 6b of the main text) that can model interactions with any functional form instead of using multiplicative interactions as in the top-SNP regression. In this section, we test the effect of each of these two components with the following two models:

1. NN SNP, which represents each gene with the top SNP but models interactions with $h_p(\cdot)$. Therefore, NN SNP removes component 1 but keeps component 2 of the proposed deep learning approach;

2. LR gene, which uses $h_{g_i}(\mathbf{X}_i; \mathbf{w}_{g_i})$ to learn the representation of each gene from the corresponding SNPs but applies linear regression with multiplicative interactions between gene representations to detect interactions. Therefore, gene LR removes component 2 but keeps component 1 of the proposed deep learning approach.

Supplementary Table 1: Ablation studies with two additional methods on two simulated datasets with complex interactions. We observe that learning the representation of each gene from the corresponding SNPs, from LR SNP to LR Gene, is especially helpful when more SNPs are causal (e.g., when the proportion of causal SNPs is 0.5). Moreover, similar to the existing XGBoost SNP approach, using NN on SNPs directly (NN SNP) does not produce good results in general.

Causal SNPs proportion	Metrics	NN	LR Gene	LR SNP	NN SNP	XGBoost SNP
0.5	AP	0.89	0.73	0.60	0.37	0.27
	AUROC	0.98	0.85	0.76	0.62	0.61
0.1	AP	0.75	0.72	0.71	0.37	0.34
	AUROC	0.88	0.85	0.85	0.66	0.71

In Supplementary Table 1, we show the results of ablation studies on two simulated datasets. We simulate the data using Equation 1 in the main text (with complex interactions), with S/N = 0.1, data size = 80k, M/I = 1.0, and two different proportions of causal SNPs, 0.5 and 0.1, respectively.

We first observe that NN SNP, which removes the gene representation learning part but keeps the complex interaction modeling part, does not work correctly in general, similar to the existing XGBoost SNP approach. We hypothesize that complex interaction models, such as NN and XGBoost, are not good at modeling Binomial distributed SNPs with tiny variances. However, the complex interaction model works properly on gene representations (e.g., in the proposed NN) which are relatively continuous.

We then observe that LR Gene, which removes the complex interaction modeling part but keeps the gene representation learning part, outperforms LR SNP, especially when more SNPs are causal in the simulator (e.g., when the proportion of causal SNPs equals 0.5). Moreover, modeling complex interactions on gene representations (from LR Gene to NN) consistently improves interaction detection in both settings because multiplication is insufficient to model the complex interactions in the simulator.

Supplementary Note 3 Additional simulation studies with simple interactions

Supplementary Note 3.1 Simulation studies with only parts of genes interacting

Similarly with experiments on simulation data with complex interactions, here we extend the experiments on data with simple interactions (Section 2.2.2) by considering only 2 genes and 5 genes out of 10 genes have simple interaction effects, such that

$$\begin{aligned} \mathbf{w}_i^{\text{main}} &= \sum_{j=1}^{d_i} \alpha_{ij}\mathbf{x}_{ij},\ \forall i \in \{1,\dots,10\}, \\ \mathbf{y} &= w_{79}\mathbf{x}_{7t_7}\mathbf{x}_{9t_9} + \sum_{k}^{10} \mathbf{w}_k^{\text{main}} + \epsilon, \end{aligned} \tag{4}$$

where only the multiplicative interaction between SNPs $\mathbf{x}_{7t_7}$ and $\mathbf{x}_{9t_9}$ is retained, and,

$$\begin{aligned} \mathbf{w}_i^{\text{main}} &= \sum_{j=1}^{d_i} \alpha_{ij}\mathbf{x}_{ij},\ \forall i \in \{1,\dots,10\}, \\ \mathbf{y} &= w_{79}\mathbf{x}_{7t_7}\mathbf{x}_{9t_9} + w_{48}\mathbf{x}_{4t_4}\mathbf{x}_{8t_8}w_{810}\mathbf{x}_{8t_8}\mathbf{x}_{10t_{10}} + \sum_{k}^{10} \mathbf{w}_k^{\text{main}} + \epsilon, \end{aligned} \tag{5}$$

where we keep 3 interactions and 5 interacting genes.

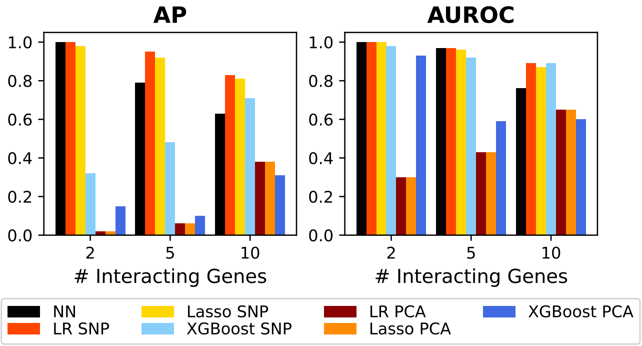

Supplementary Figure 17: Comparison of gene-gene interaction detection methods in terms of AP and AUROC on datasets with simple interactions. We simulate data using S/N = 0.1, data size = 80k, prop causal SNPs = 50%, M/I = 1.0, and three different number of interacting genes: 2, 5, and 10 (simulator shown in the main text). Regression with top-SNP are better than others in general, except when only 2 genes are interacting where the NN approach (black bar) outperforms others.

From Supplementary Figure 17, we observe that the top-SNP regression approaches are better than NNs in general, when interactions are simple.

Supplementary Note 3.2 Simulation studies with more genes

Here we scale up the experiments in Section 2.2.2 by considering 50 and 100 genes instead of only 10. We consider the following simulator:

$$
\begin{aligned}
\mathbf{w}_i^{\text{main}} &= \sum_{j=1}^{d_i} \alpha_{ij}\mathbf{x}_{ij},\ \forall i \in \{1,\dots,10A\}, \\
\mathbf{y} &= \sum_{a=0}^{A-1} (w_{12}\mathbf{x}_{1t_1}\mathbf{x}_{2t_2} + w_{79}\mathbf{x}_{7t_7}\mathbf{x}_{9t_9} + w_{35}\mathbf{x}_{3t_3}\mathbf{x}_{5t_5} + w_{48}\mathbf{x}_{4t_4}\mathbf{x}_{8t_8} + w_{67}\mathbf{x}_{6t_6}\mathbf{x}_{8t_8} + w_{810}\mathbf{x}_{8t_8}\mathbf{x}_{10t_{10}}) \\
&\quad + \sum_{k}^{10A} \mathbf{w}_k^{\text{main}} + \epsilon,
\end{aligned} \tag{6}
$$

with $A = 5$ and $A = 10$. In the above simulator, all genes have interaction effects, and the number of interactions are scaled up according to the number of genes.

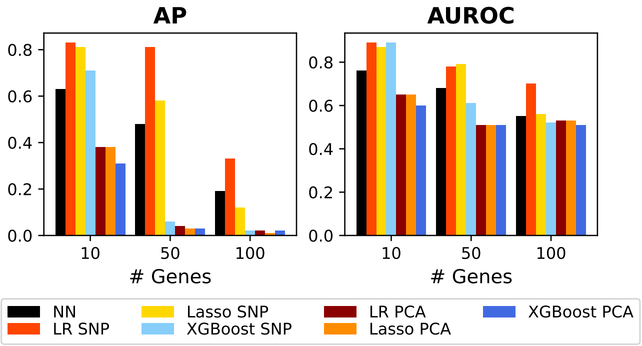

Supplementary Figure 18: Comparison of gene-gene interaction detection methods in terms of AP and AUROC on datasets with simple interactions. We simulate data using S/N = 0.1, data size = 80k, prop causal SNPs = 50%, M/I = 1.0, and three different number of genes: 10 (simulator shown in the main text), 50, and 100. Linear regression and Lasso with top SNPs outperform others when interactions are simple.

Similar with Supplementary Figure 4, we observe that as the number of genes of the simulator increases, the performance of all methods drops in Supplementary Figure 18, because the total signal-to-noise ratio is fixed and the effect of each interaction decreases when more genes and interactions are included in the simulator. LR with top-SNP methods are better than others especially when number of genes is huge and interactions between genes are simple.

Supplementary Note 4 Additional simulation studies without interactions

Supplementary Note 4.1 Calibration plot of existing permutation methods

We show the calibration plot of current permutation approaches (Perm T and Perm R) in Supplementary Figure 19. We observe that both permutation approaches cannot generate calibrated null distributions.

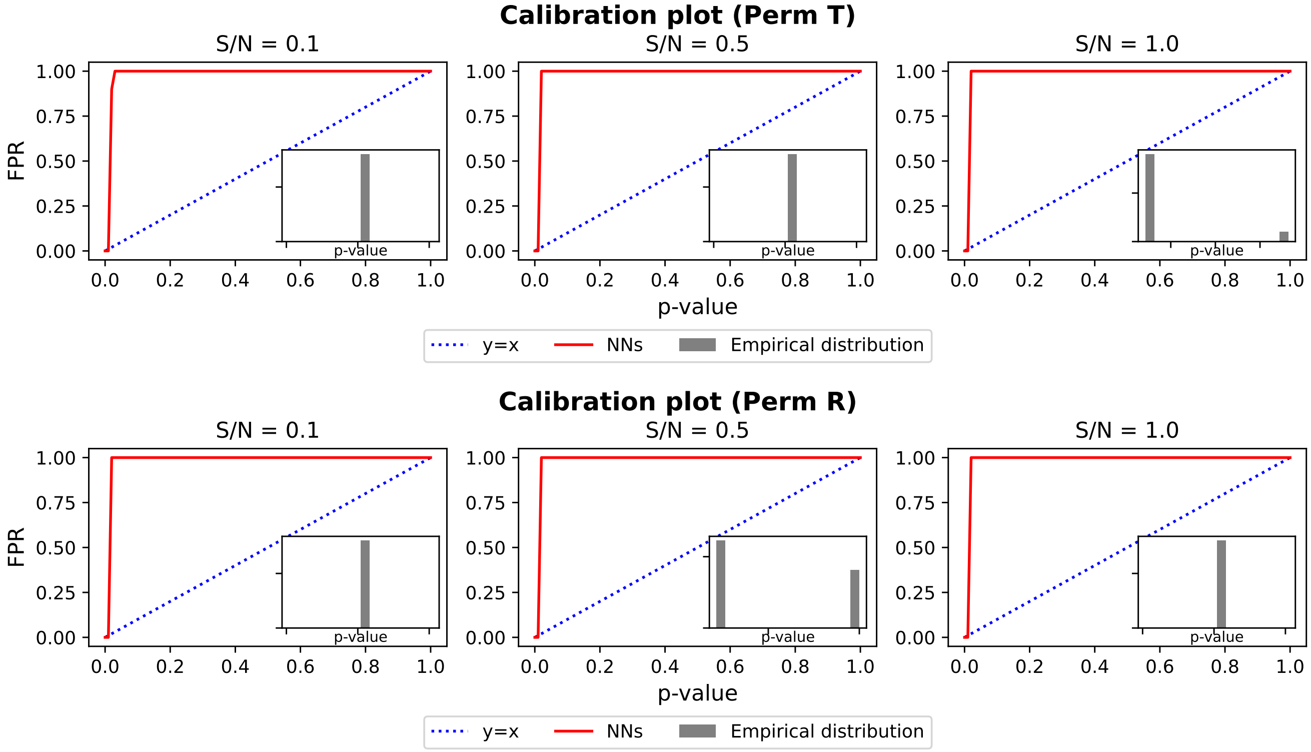

Supplementary Figure 19: Calibration plots of the p-values from the NNs interactions with two existing permutation test (Perm T and Perm R) on 20 null simulation datasets for each setting. We observe that two permutation approaches are not well calibrated.

Supplementary Note 4.2 Permutation approach for NNs with maxT correction

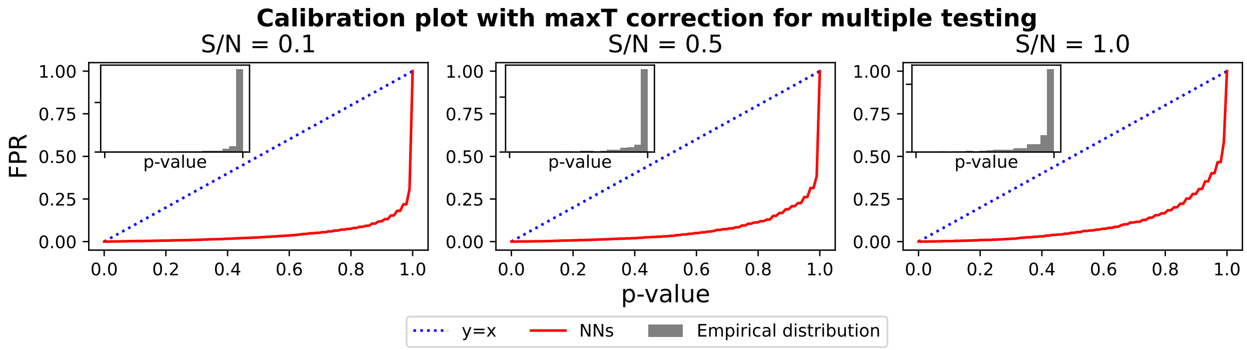

Supplementary Figure 20: Calibration plots of the novel permutation procedure for the NNs interactions on 20 null simulations with the maxT multiple testing correction. We observe that using the p-value corrected by maxT can control FPR well.

Supplementary Note 5 Visualization of replicable interactions

We visualize another replicable interactions and the corresponding fits for the linear regressions for each phenotype in Supplementary Figure 21-23.

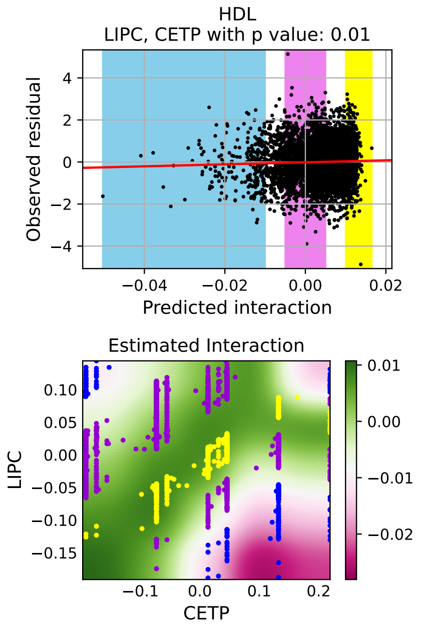

Supplementary Figure 21: Replicate interactions on HDL detected from UK Biobank on FINRISK dataset. **Top**: Visualization of linear regressions for replication. **Bottom**: Visualization of corresponding gene-gene interactions, where the heatmap represent the learned interaction function.

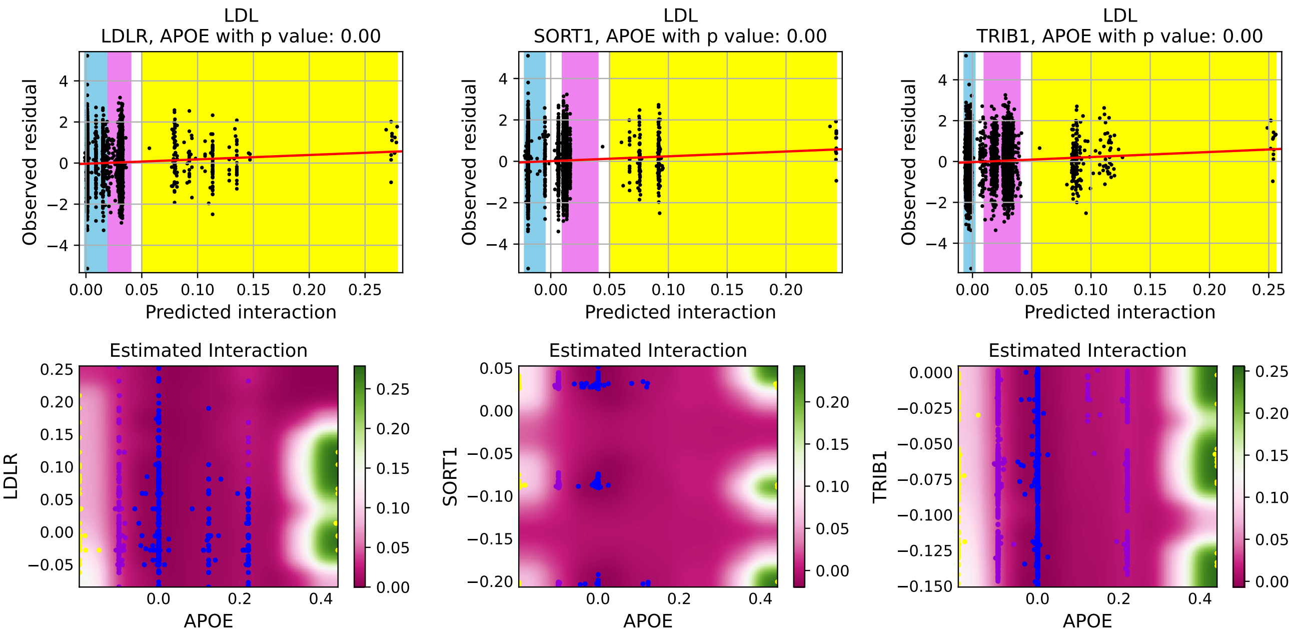

Supplementary Figure 22: Replicate interactions on LDL detected from UK Biobank on FINRISK dataset. **Top**: Visualization of linear regressions for replication. **Bottom**: Visualization of corresponding gene-gene interactions, where the heatmap represent the learned interaction function.

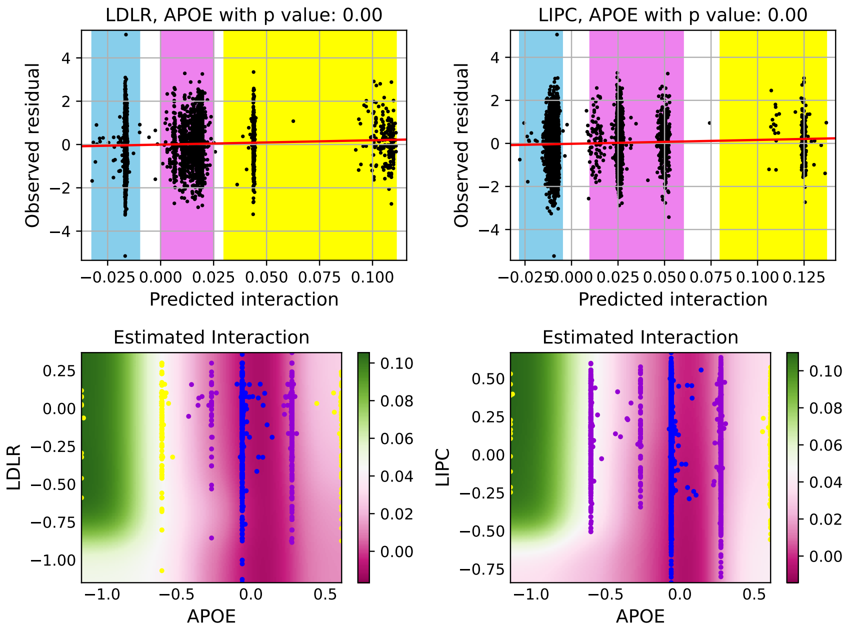

Supplementary Figure 23: Replicate interactions on TC detected from UK Biobank on FINRISK dataset. **Top**: Visualization of linear regressions for replication. **Bottom**: Visualization of corresponding gene-gene interactions, where the heatmap represent the learned interaction function.

Supplementary Note 6 Additional experiments on UK Biobank

Supplementary Note 6.1 Compare the R^2 of the top-SNP approach and NNs

In Supplementary Table 2, we compute the test set proportional of variance explained (R^2) of linear regression with the multiplicative interaction term with both top-SNP representations and gene representations learned from NNs for each candidate gene, as well as the R^2 of neural network with learned gene representations.

We notice that although using neural networks can improve the variance explained, the sizes of increases from Gene LR to NN are generally small. However, the increases from top-SNP LR to NN are large. This indicates that the benefits of NNs are mainly from using all possible SNPs from each gene to learn the gene representation.

Supplementary Table 2: Proportion of variance explained (R^2) by linear regressions with both top-SNP and gene representations, and neural networks on each pair of interactions. We observe that the R^2 from top-SNP LR to NNs is much higher than from Gene LR to NNs.

Phenotype	Genes	Top-SNP LR, test R^2	Gene LR, test R^2	NN, test R^2	test R^2 increases Top-SNP LR $\rightarrow$ NN	test R^2 increases Gene LR $\rightarrow$ NN
HDL	*LPL*, *CETP*	0.0333	0.0526	0.0528	0.580	0.002
	LIPC, *CETP*	0.0323	0.0525	0.0528	0.629	0.003
	CETP, *LIPG*	0.0287	0.0450	0.0452	0.567	0.002
	ABCA1, *CETP*	0.0284	0.0456	0.0458	0.604	0.002
	LPL, *LIPC*	0.0128	0.0269	0.0270	1.098	0.003
	LIPC, *LIPG*	0.009	0.0194	0.0194	1.075	-0.001
LDL	*LDLR*, *APOE*	0.0399	0.0485	0.0491	0.226	0.009
	SORT1, *APOE*	0.0381	0.0433	0.0439	0.146	0.009
	LPA, *APOE*	0.0342	0.0382	0.0387	0.129	0.011
	TRIB1, *APOE*	0.0344	0.0398	0.0404	0.169	0.010
	HMGCR, *APOE*	0.0350	0.0402	0.0407	0.158	0.010
	APOB, *APOE*	0.0344	0.0427	0.0432	0.254	0.009
	PCSK9, *APOE*	0.0369	0.0382	0.0387	0.044	0.010
TC	*LDLR*, *APOE*	0.0259	0.0323	0.0333	0.281	0.029
	PCSK9, *APOE*	0.0230	0.0277	0.0287	0.246	0.033
	SORT1, *APOE*	0.0236	0.0269	0.0279	0.178	0.033
	LIPG, *APOE*	0.0203	0.0245	0.0257	0.256	0.039
	LIPC, *APOE*	0.0211	0.0261	0.0271	0.284	0.034
	APOE, *APOC1*	0.0243	0.0254	0.0253	0.040	-0.001

Supplementary Note 6.2 Compare the interaction detection power of the top-SNP approach and NNs

In Supplementary Figure 24, we train a bivariate LR with a multiplicative interaction term for each interaction pair, where the gene is represented by the corresponding top SNP. We collect the negative log p-value (y axis) of the multiplicative interaction term of linear regression and the corresponding gene-gene interaction score (x axis) ofrom NNs for each interaction terms.

We apply two FDR (false discovery rate) thresholds: 0.1 and 0.3. We observe that NNs can find very different interactions from the top-SNP approach, and for a certainty threshold, the number of findings from NNs is smaller than top-SNP approach. This indicate that most interactions in real-world applications (e.g., UK Biobank dataset) can be approximated well by multiplications between top SNPs, and then that is the best thing to do. Therefore the NN approach should not be used as a substitute for the default top-SNP approach, but rather as a complementary tool to find interactions that would be missed otherwise.

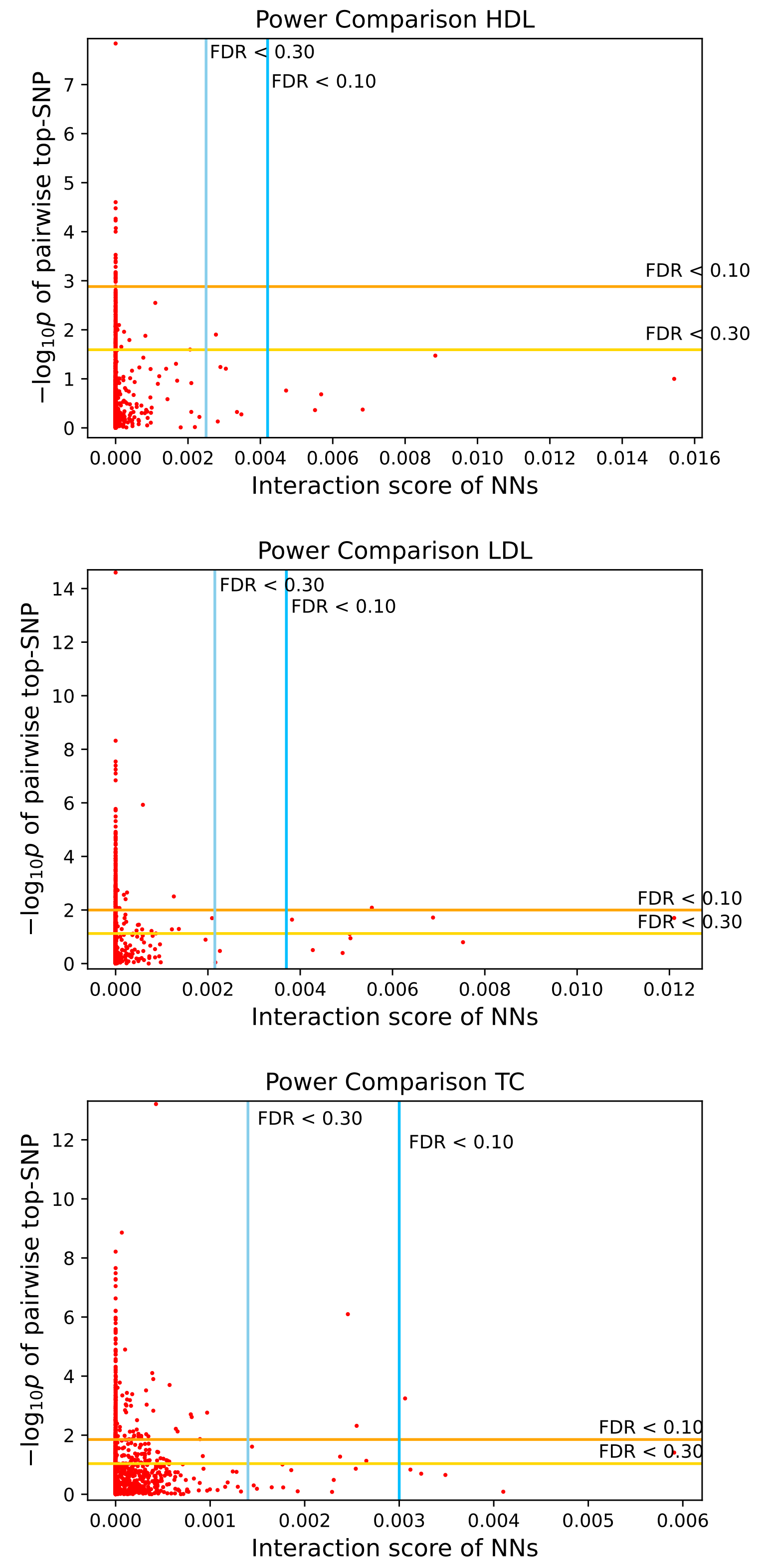

Supplementary Figure 24: Compare the findings of top-SNP approach and NNs approach with same FDR thresholds for each phenotype. Each red dot represents one interaction pair, and the corresponding x and y coordinates are the interaction score from NNs and the negative log p-value of top-SNP regression respectively. We notice that NNs can find very different interactions from the default top-SNP regression approach, and the number of findings are smaller than top-SNP approach with the same FDR threshold.
