# Supplementary material for: Gene–gene interaction detection with deep learning
Source: Commun Biol. 2022 Nov 12;5:1238. doi: 10.1038/s42003-022-04186-y (PMC9653457; doi:10.1038/s42003-022-04186-y)
Supplement: Supplementary file 3 — Description of Additional Supplementary Files [file 42003_2022_4186_MOESM3_ESM.pdf]

Description of Additional Supplementary Files

File name: Supplementary Data 1

Description: The source data behind the graphs in the paper.
