# Supplementary material for: Gene–gene interaction detection with deep learning
Source: Commun Biol. 2022 Nov 12;5:1238. doi: 10.1038/s42003-022-04186-y (PMC9653457; doi:10.1038/s42003-022-04186-y)
Supplement: Supplementary file 5 — Reporting Summary [file 42003_2022_4186_MOESM5_ESM.pdf]

nature portfolio

Corresponding author(s): Tianyu Cui

Last updated by author(s): Oct 21, 2022

Reporting Summary

Nature Portfolio wishes to improve the reproducibility of the work that we publish. This form provides structure for consistency and transparency in reporting. For further information on Nature Portfolio policies, see our Editorial Policies and the Editorial Policy Checklist.

Statistics

For all statistical analyses, confirm that the following items are present in the figure legend, table legend, main text, or Methods section.

n/a	Confirmed	
☐	☒	The exact sample size (*n*) for each experimental group/condition, given as a discrete number and unit of measurement
☐	☒	A statement on whether measurements were taken from distinct samples or whether the same sample was measured repeatedly
☐	☒	The statistical test(s) used AND whether they are one- or two-sided<br>*Only common tests should be described solely by name; describe more complex techniques in the Methods section.*
☐	☒	A description of all covariates tested
☐	☒	A description of any assumptions or corrections, such as tests of normality and adjustment for multiple comparisons
☐	☒	A full description of the statistical parameters including central tendency (e.g. means) or other basic estimates (e.g. regression coefficient) AND variation (e.g. standard deviation) or associated estimates of uncertainty (e.g. confidence intervals)
☐	☒	For null hypothesis testing, the test statistic (e.g. *F*, *t*, *r*) with confidence intervals, effect sizes, degrees of freedom and *P* value noted<br>*Give P values as exact values whenever suitable.*
☒	☐	For Bayesian analysis, information on the choice of priors and Markov chain Monte Carlo settings
☒	☐	For hierarchical and complex designs, identification of the appropriate level for tests and full reporting of outcomes
☐	☒	Estimates of effect sizes (e.g. Cohen's *d*, Pearson's *r*), indicating how they were calculated

Our web collection on statistics for biologists contains articles on many of the points above.

Software and code

Policy information about availability of computer code

Data collection	No software was used
Data analysis	We use QCTOOL v2.0.7 for quality control; We provide the source code of the method: https://github.com/tycui/GWAS_NN

For manuscripts utilizing custom algorithms or software that are central to the research but not yet described in published literature, software must be made available to editors and reviewers. We strongly encourage code deposition in a community repository (e.g. GitHub). See the Nature Portfolio guidelines for submitting code & software for further information.

Data

Policy information about availability of data

All manuscripts must include a data availability statement. This statement should provide the following information, where applicable:

- Accession codes, unique identifiers, or web links for publicly available datasets
- A description of any restrictions on data availability
- For clinical datasets or third party data, please ensure that the statement adheres to our policy

UK Biobank data are available to registered investigators under approved applications (http://www.ukbiobank.ac.uk); A permission to use the FINRISK data can be applied from THL (Finnish Institute for Health and Welfare); Other relevant data are available from the corresponding author upon request.

Field-specific reporting

Please select the one below that is the best fit for your research. If you are not sure, read the appropriate sections before making your selection.

☒ Life sciences ☐ Behavioural & social sciences ☐ Ecological, evolutionary & environmental sciences

For a reference copy of the document with all sections, see nature.com/documents/nr-reporting-summary-flat.pdf

Life sciences study design

All studies must disclose on these points even when the disclosure is negative.

Sample size	We use all samples available in UK Biobank (N=424,389) and FINRISK (N=4,620).
Data exclusions	We remove SNPs from the genetic data that do not pass the quality control, i.e., missing rate > 0.05, MAF < 0.01, pHWE < 10^{-6} and info score (imputation quality) < 0.4. We analyse interactions only between 65 genes with previously identified main effects on cholesterol (selecting genes based on their main effects does not bias the assessment of the significance of interactions using permutation).
Replication	We replicate interactions detected on the UK Biobank by testing if a similar effect exists in an independent dataset FINRISK. Out of 19 possible interactions, 9 interactions replicate significantly in FINRISK. Possible reasons for interactions that do not replicate: 1. small sample size of the FINRISK data; 2. different cohorts have different effect sizes.
Randomization	We randomly sample 70% individuals from the dataset for training and 30% for testing.
Blinding	Investigators are blinded to group allocation.

Reporting for specific materials, systems and methods

We require information from authors about some types of materials, experimental systems and methods used in many studies. Here, indicate whether each material, system or method listed is relevant to your study. If you are not sure if a list item applies to your research, read the appropriate section before selecting a response.

Materials & experimental systems

n/a	Involved in the study	
☒	☐	Antibodies
☒	☐	Eukaryotic cell lines
☒	☐	Palaeontology and archaeology
☒	☐	Animals and other organisms
☒	☐	Human research participants
☒	☐	Clinical data
☒	☐	Dual use research of concern

Methods

n/a	Involved in the study	
☒	☐	ChIP-seq
☒	☐	Flow cytometry
☒	☐	MRI-based neuroimaging
